# mRNA-1273 boost after BNT162b2 vaccination generates comparable SARS-CoV-2-specific functional responses in naïve and COVID-19-recovered individuals

**DOI:** 10.3389/fimmu.2023.1136029

**Published:** 2023-04-21

**Authors:** Roberto Lozano-Rodríguez, José Avendaño-Ortíz, Verónica Terrón, Karla Montalbán-Hernández, José Casalvilla-Dueñas, Marta Bergón-Gutiérrez, Pablo Mata-Martínez, Alejandro Martín-Quirós, Miguel Ángel García-Garrido, Álvaro del Balzo-Castillo, María Peinado, Laura Gómez, Irene Llorente-Fernández, Gema Martín-Miguel, Carmen Herrero-Benito, Lissette López-Morejón, Carmen Vela-Olmo, Carolina Cubillos-Zapata, Eduardo López-Collazo, Carlos del Fresno

**Affiliations:** ^1^ The Innate Immune Response Group, Hospital la Paz Institute for Health Research (IdiPAZ), La Paz University Hospital, Madrid, Spain; ^2^ Tumor Immunology Laboratory, Hospital la Paz Institute for Health Research (IdiPAZ), La Paz University Hospital, Madrid, Spain; ^3^ Immunomodulation Laboratory, Hospital la Paz Institute for Health Research (IdiPAZ), La Paz University Hospital, Madrid, Spain; ^4^ Emergency Department and Emergent Pathology Research Group, Hospital la Paz Institute for Health Research (IdiPAZ), La Paz University Hospital, Madrid, Spain; ^5^ Intensive Care Unit, Infanta Cristina University Hospital, Parla, Madrid, Spain; ^6^ Pediatric Intensive Care Unit, 12 de Octubre University Hospital, Madrid, Spain; ^7^ Eurofins-Ingenasa, Madrid, Spain; ^8^ Centro de Investigación Biomédica en Red (CIBER) of Respiratory Diseases (CIBERES), Madrid, Spain

**Keywords:** mRNA vaccine, COVID-19 booster, SARS-CoV-2-specific, naïve, recovered from COVID-19

## Abstract

**Introduction:**

COVID-19 vaccines based on mRNA have represented a revolution in the biomedical research field. The initial two-dose vaccination schedule generates potent humoral and cellular responses, with a massive protective effect against severe COVID-19 and death. Months after this vaccination, levels of antibodies against SARS-CoV-2 waned, and this promoted the recommendation of a third vaccination dose.

**Methods:**

We have performed an integral and longitudinal study of the immunological responses triggered by the booster mRNA-1273 vaccination, in a cohort of health workers previously vaccinated with two doses of the BNT162b2 vaccine at University Hospital La Paz located in Madrid, Spain. Circulating humoral responses and SARS-CoV-2-specific cellular reactions, after *ex vivo* restimulation of both T and B cells (cytokines production, proliferation, class switching), have been analyzed. Importantly, all along these studies, the analyses have been performed comparing naïve and subjects recovered from COVID-19, addressing the influence of a previous infection by SARS-CoV-2. Furthermore, as the injection of the third vaccination dose was contemporary to the rise of the Omicron BA.1 variant of concern, T- and B-cell-mediated cellular responses have been comparatively analyzed in response to this variant.

**Results:**

All these analyses indicated that differential responses to vaccination due to a previous SARS-CoV-2 infection were balanced following the boost. The increase in circulating humoral responses due to this booster dropped after 6 months, whereas T-cell-mediated responses were more stable along the time. Finally, all the analyzed immunological features were dampened in response to the Omicron variant of concern, particularly late after the booster vaccination.

**Conclusion:**

This work represents a follow-up longitudinal study for almost 1.5 years, analyzing in an integral manner the immunological responses triggered by the prime-boost mRNA-based vaccination schedule against COVID-19.

## Introduction 

Pandemic COVID-19 has impacted worldwide to an unprecedented depth in modern times. The lockdown of entire countries, population confinement, and social distance policies were measures not even imagined before this global crisis (1). Biomedical science has responded to this challenge with the development of effective vaccines that have allowed to recover most of the regular habits known before December 2019. Among these vaccines, mRNA-based jabs have been developed and administered for the first time to human beings, with two different options such as BNT162b2 (Pfizer-BioNTech) and mRNA-1273 (Moderna-NIAID) vaccines conferring protection against severe forms of COVID-19 (2). This is due to the triggering of combined B-cell-based humoral responses, along with cellular reactions mediated by T cells. Interestingly, although the relevance of neutralizing antibodies is clear, till which extent both branches of immunity contribute to the actual response against the infection along the time and against the COVID-19 pathology is still a matter of debate. Along these lines, it is important to highlight the importance of an adequate assessment of immunological responses ignited against both SARS-CoV-2 infection and COVID-19 vaccines (3, 4), as this might impact clinically relevant interpretations.

These brand-new mRNA-based vaccines were designed to be administered in a two-dose regimen. We and others have studied the evolution of responses ignited by this vaccination schedule along the time (5, 6). There is a quite established consensus about the generation of potent humoral and cellular responses early after vaccination that decayed after 6–8 months (7). Contemporarily to this waning, a raise of infections due to SARS-CoV-2 variants of concerns (VoC) took place, which, at first, alarmed about till which extent initial vaccines would achieve a proper coverage against them (8). These two main factors accelerated the recommendation for a third vaccination dose or boost. Indeed, studies including large cohorts such as the ZEO COVID study indicated that booster doses restore vaccine effectiveness waned after the second dose, no matter the vaccine initially administered (9). At this point, heterologous vaccination boosts as the one studied in this work, meaning the administration of a third vaccination with a different vaccine than the one administered during the prime phase, showed stronger immunological responses (10, 11). This was accompanied by a reduced incidence of SARS-CoV-2-confirmed infections in individuals receiving heterologous compared with homologous boosting (12). Notably, slight differences observed between the two mRNA vaccines after the two-dose prime phase were balanced with the booster dose (13). Interestingly, the best sequence of heterologous prime-boost schedule appears to be the combination of mRNA vaccines, even against variants of concern (14). Note that in these studies, previous infections by SARS-CoV-2 influenced the responses triggered by prime vaccination, with cellular and humoral reactions differing between naïve and subjects recovered from COVID-19 (5), even after only a single dose of the mRNA vaccine (15–17). Interestingly, strong hybrid immunity due to SARS-CoV-2 infection and the complete initial full vaccination was detected, no matter whether the infection was before or after vaccination (18). Notably, differential immunological patterns observed between the four EMA-approved vaccines against COVID-19 appeared balanced by a prior infection with SARS-CoV-2 (19).

Nevertheless, although it is interesting to address whether responses triggered after the vaccination boost are also influenced by a previous infection with SARS-CoV-2, comparative information of putative differential responses triggered by booster vaccines in naïve and COVID-19 convalescent individuals is scarce (20), most likely due to the growing difficulty to find participants not previously infected. In this work, we hypothesize that a former infection with SARS-CoV-2 might generate differential responses triggered by the booster vaccine dose.

Considering all these factors, the rising in the incidence in the current autumn and winter, along with the beginning of the administration of a fourth booster dose, in here we have addressed the immunological responses triggered by a heterologous prime-boost regime with BNT162b2 plus mRNA-1273 boost, in a longitudinal cohort for almost 1.5 years. This work represents an integral study of such immunological features including circulating antibodies and T cell- and B cell-mediated cellular responses, against both the wild-type (WT) and Omicron BA.1 (B.1.1.529) VoC, differentiating between naïve and COVID-19-recovered individuals. We believe this is a timely study covering critical immunological features triggered after vaccination, including some not commonly analyzed such as B-cell class-switching and B-cell antigen-specific cytokine production after restimulation, to fully understand protective responses ignited by mRNA-based COVID-19 heterologous vaccination in the long time.

## Material and methods

### Longitudinal sampling of healthy medical personnel volunteers

**Figure 1:**
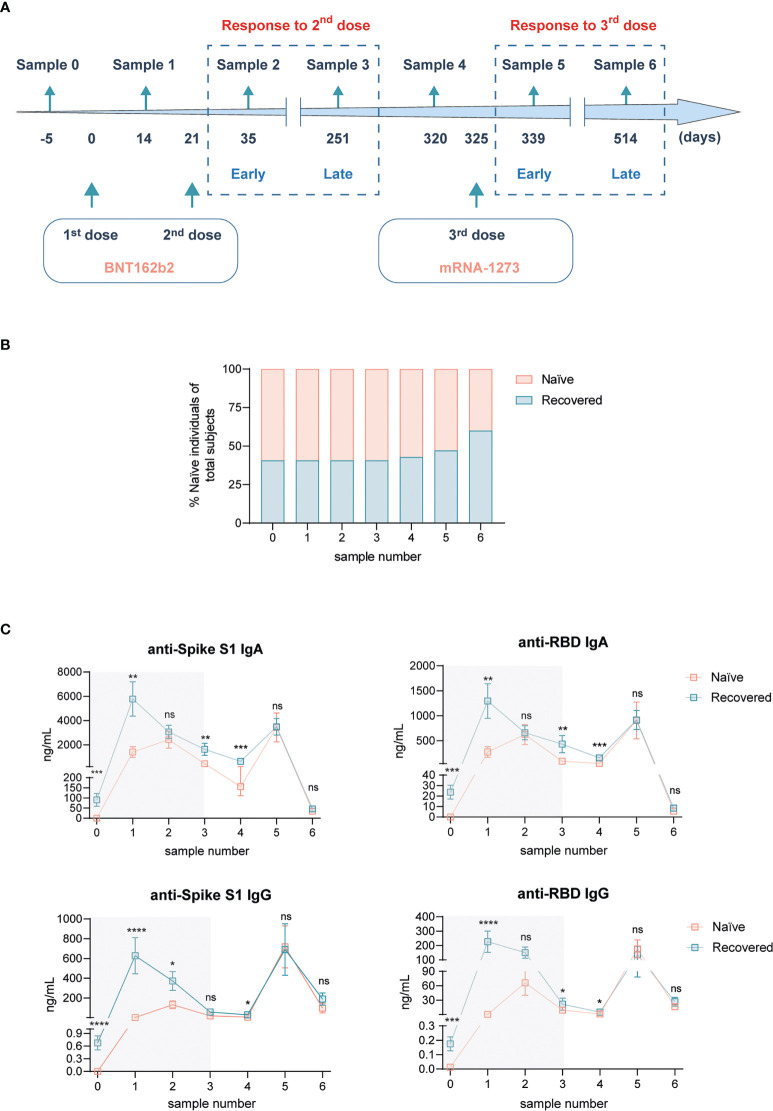
Humoral response Spike-specific of SARS-CoV-2 subsequent to BNT162b2 mRNA vaccination plus mRNA-1273 booster dose in naïve individuals and individuals recovered from COVID-19. **(A)** Experimental design. Blood samples from healthy medical personnel were collected 5 days before the first dose of BNT162b2 mRNA vaccine (sample 0), 14 days after the first dose of BNT162b2 mRNA vaccine (sample 1), 14 days (sample 2, 35 days from the beginning), 230 days (sample 3, 251 days from the beginning), and 69 days (sample 4, 320 days from the beginning) after the second dose of BNT162b2 mRNA vaccine. Subsequent blood samples were collected 14 days (sample 5, 339 days from the beginning) and 189 days (sample 6, 514 days from the beginning) following the administration of the mRNA-1273 booster dose. **(B)** Frequency of naïve participants and recovered from COVID-19 along the study. **(C)** Plasmatic levels of anti-Spike S1 IgA (upper left panel), anti-receptor binding domain (RBD) IgA (upper right panel), anti-Spike S1 IgG (lower left panel), and anti-RBD IgG (lower right panel) antibodies. Shadowed areas represent analyses performed in (5). n = 16 naïve, n = 11 recovered from COVID-19 at sample 0. Data in **(C)** are shown as mean ± SEM. Unpaired Student’s *t-*test in each sample number between naïve and COVID-19-recovered subjects (ns, not significant; *p < 0.05; **p < 0.01; ***p < 0.001; ****p < 0.0001). In every plot, all the possible experimental conditions were compared; statistically significant differences are indicated, and the lack of statistical indication means the absence of a significant difference.

Blood samples of 27 healthy medical personnel volunteers of the University Hospital La Paz Institute for Health Research in Madrid (Spain) were collected for this study before and after the vaccination against Spike protein of SARS-CoV-2 according to two doses of BNT162b2 (30 μg) followed by a booster dose of mRNA-1273 (50 μg). Samples were retrieved at seven different time points: 5 days before the first dose of BNT162b2 vaccine (sample 0, N = 27, naïve = 16, recovered = 11), 14 days after the first dose of BNT162b2 vaccine (sample 1, N = 27, naïve = 16, recovered = 11), 14 days after the second dose of BNT162b2 vaccine (sample 2, N = 27, naïve = 16, recovered = 11), 230 days after the second dose of BNT162b2 vaccine (sample 3, N = 27, naïve = 16, recovered = 11), 299 days after the second dose of BNT162b2 vaccine and 5 days before the booster dose of mRNA-1273 vaccine (sample 4, N = 21, naïve = 11, recovered = 10), 318 days after the second dose of BNT162b2 vaccine and 14 days after the booster dose of mRNA-1273 vaccine (sample 5, N = 17, naïve = 9, recovered = 8), and 493 days after the second dose of BNT162b2 vaccine and 189 days after the booster dose of mRNA-1273 vaccine (sample 6, N = 20, naïve = 8, recovered = 12) (**Figure 1A**). Note that all the participants were gathered exactly at the indicated time points to collect their blood samples. According to the ethical guidelines of the 1975 Declaration of Helsinki, we collected the informed consent from all healthy medical personnel volunteers in obedience with the ethical standards stablished. The study was authorized by the La Paz University Hospital Research Ethics Committee (PI-4100).

### PBMC isolation procedure and culture conditions

Peripheral blood mononuclear cells (PBMCs) from BNT162b2 two-dose- and mRNA-1273 booster-vaccinated healthy medical personnel volunteers were obtained from venous blood with EDTA anticoagulant, using Ficoll-Plus (GE Healthcare Bio-Sciences, Chicago, IL) solution based on the manufacturer’s instructions. PBMCs were counted using Trypan blue staining after washing them twice with phosphate-buffered saline (PBS). Before some stimulations such as activation or proliferation, the culture of fresh PBMCs was plated in RPMI 1640 medium supplemented with 10% fetal bovine serum (FBS), 2 mM L-glutamine, 25 mM HEPES, and 1% streptomycin and penicillin mix (Gibco, Billings, MT). A humidified incubator at 37°C at 5% CO_2_ was used to culture fresh PBMCs.

### Plasma collection

Plasma samples from healthy medical personnel vaccinated with BNT162b2 two-dose and mRNA-1273 boosters were obtained from venous blood with EDTA anticoagulant using a density gradient of Ficoll-Plus solution following the typical centrifugation method. Subsequently, they were aliquoted and stored at -80°C until use. 

### Anti-SARS-CoV-2 IgA and IgG antibody quantification

For the quantification of specific IgA and IgG antibodies against Spike protein of SARS-CoV-2, saved frozen plasma samples from healthy medical personnel vaccinated with BNT162b2 two-dose and mRNA-1273 boosters were thawed. Prior to use, all plasma samples were centrifuged at 1,000 relative centrifugal force (rcf) for 30 min to remove possible debris. Two bead-based immunoassays, both from BioLegend (San Diego, CA), the LEGENDplex SARS-CoV-2 Serological IgA Panel (2-plex, Spike (S1) and receptor binding domain (RBD) of Spike protein) and LEGENDplex SARS-CoV-2 Serological IgG Panel (3-plex, Spike (S1), receptor binding domain (RBD) of Spike protein and nucleocapsid (N)), were used following the manufacturer’s instructions to quantify the titers of IgA and IgG antibodies, respectively, in plasma samples. A FACSCalibur flow cytometer (BD Biosciences, Franklin Lakes, NJ) were used to acquire the samples, and data were analyzed by LEGENDplex (BioLegend) v.8 software. The presence of anti-SARS-CoV-2 N immunoglobulins, indicative of a former SARS-CoV-2 viral infection, was confirmed by the CE-IVD-marked system INgezim COVID 19 DR50.CoV.KO provided by Eurofins Ingenasa.

### Supernatant collection and SARS-CoV-2 Spike-specific T-cell proliferation assays

PBMCs from healthy medical personnel, 14 (sample 2) and 230 (sample 3) days after the second dose of the BNT162b2 vaccine, and 14 (sample 5) and 189 (sample 6) after the booster dose of the mRNA-1273 vaccine, were obtained from venous blood with EDTA anticoagulant using Ficoll-Plus based on the manufacturer’s instructions. PBMCs were counted using Trypan blue staining after washing them twice with phosphate-buffered saline (PBS) and cultured in RPMI 1640 medium supplemented with 10% fetal bovine serum (FBS), 2 mM L-glutamine, 25 mM HEPES, and 1% penicillin and streptomycin mix. To evaluate the T lymphocyte proliferation, PBMCs were stained with carboxyfluorescein succinimidyl ester (CFSE) (Thermo Fisher Scientific, Waltham, MA) following the manufacturer’s protocol. Next, CFSE-labeled PBMCs were plated in a 96-well flat-bottom plate (1.5 × 10^6^ cells/well) in RPMI 1640 medium supplemented with 10% fetal bovine serum (FBS), 2 mM L-glutamine, 25 mM HEPES, and 1% penicillin and streptomycin mix. CFSE-labeled PBMCs of samples 2, 3, 5, and 6 were stimulated or not with PepTivator SARS-CoV-2 Prot_S (Miltenyi Biotec, Bergisch Gladbach, Germany) for 5 days to allow T-cell proliferation at 37°C at 5% CO_2_, whereas samples 5 and 6 were also stimulated or not with PepTivator SARS-CoV-2 Prot_S B.1.1.529/BA.1 wild-type reference pool or PepTivator SARS-CoV-2 Prot_S B.1.1.529/BA.1 mutation pool (both from Miltenyi Biotec) for 5 days to allow T-cell proliferation at 37°C at 5% CO_2_. Note that both *PepTivator SARS-CoV-2 Prot_S* and *PepTivator SARS-CoV-2 Prot_S B.1.1.529/BA.1 wild-type reference pool* represent the sequence of the WT/Wuhan SARS-CoV-2 strain, nevertheless covering different regions of the Spike protein. Subsequently to the proliferation assay, supernatants were taken, aliquoted, and stored at -80°C until use. For the PBMC staining, PBMCs were washed with PBS and stained with fluorochrome-conjugated antibodies against surface markers listed in **Supplementary Table 1**. LIVE/DEAD Blue fluorescent reactive dye purchased from Invitrogen was used to exclude the debris and dead cells. To avoid the non-specific binding of certain fluorochromes on monocytes, the True-Stain Monocyte Blocker (BioLegend) reagent was added prior to the label protocol. A Cytek Aurora Spectral Cytometer (Cytek Biosciences, Fremont, CA) were used to acquire the labeled cells, and data were analyzed using FlowJo (TreeStar, Ashland, OR) v10.6.2 software.

### Antibody-secreting cell generation and their functional assessment against wild-type and BA.1 Omicron variant Spike SARS-CoV-2 proteins

PBMCs from healthy medical personnel 493 days after the second dose of BNT162b2 vaccine and 189 days after the booster dose of mRNA-1273 SARS-CoV-2 mRNA vaccine (sample 6) were obtained from venous blood with EDTA anticoagulant using Ficoll-Plus following the manufacturer’s instructions. PBMCs were counted using Trypan blue staining after washing them twice with phosphate-buffered saline (PBS). Following the PBMC counting, 6 × 10^6^ PBMCs were plated in a 24-well flat-bottom plate (1.5 × 10^6^ cells/well) in RPMI 1640 medium supplemented with 10% fetal bovine serum (FBS), 2 mM L-glutamine, 25 mM HEPES, and 1% penicillin and streptomycin mix. They were stimulated with IL-4 (70 ng/ml, PeproTech, Cranbury, NJ) and an agonistic mouse anti-human CD40 (5 μg/ml, BioGems, Seoul, South Korea) for 6 days to perform a polyclonal stimulation of memory B cells (MBCs). High-binding 96-well plates were prehydrated with 150 μl of PBS and then coated with 50 μl of bovine serum albumin (BSA, 5 μg/ml), the recombinant wild-type SARS-CoV-2 spike full protein ECD (5 μg/ml, Sino Biological, Beijing, China), or the recombinant B.1.1.529/BA.1 Omicron variant of SARS-CoV-2 spike full protein ECD (5 μg/ml, Sino Biological) overnight at 4°C. Supernatants were removed from the coated wells and washed twice with PBS and blocked with RPMI 1640 medium supplemented with 10% fetal bovine serum (FBS), 2 mM L-glutamine, 25 mM HEPES, and 1% penicillin and streptomycin mix for 1 h at 37°C at 5% CO_2_ in a humidified incubator. After that, in a volume of 200 µl/well, 1.5 × 10^5^ polyclonal stimulated memory B cells were seeded to evaluate the functional spike-specific memory B-cell response for 44 h at 37°C at 5% CO_2_. After the functional Spike-specific memory B-cell assay, supernatants were obtained, aliquoted, and stored at -80°C until use. Cells were recovered and stained for surface markers with fluorochrome-conjugated antibodies listed in **Supplementary Table 2**.

### Cytokine quantification in supernatants

Based on a bead-based immunoassay, LEGENDplex Human Essential Immune Response Panel (13-plex: IL-1β, IL-2, IL-4, IFNγ, TNF-α, MCP-1 (CCL2), CXCL10, IL-6, IL-8 (CXCL8), IL-10, IL-12p70, IL-17A, and free active TGF-β1) was used to quantify the concentration of cytokines in supernatant samples from both T and B Spike-specific stimulations, following the manufacturer’s instructions. A FACSCalibur flow cytometer was used to acquire the samples, and data were analyzed by the LEGENDplex v.8 software. 

### Statistical analysis of biological data

**Figure 2:**
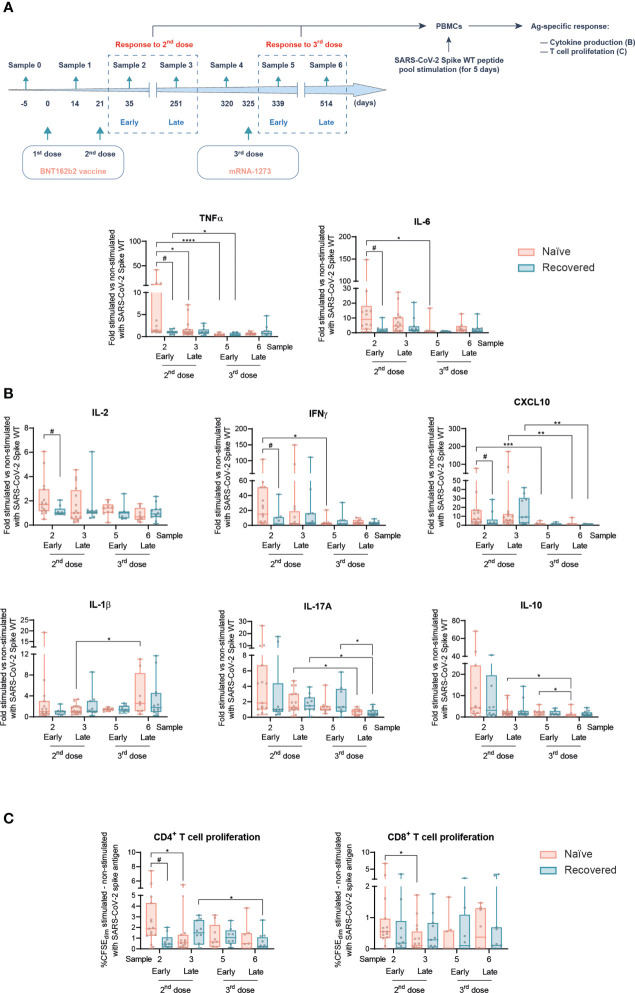
*Ex vivo* T cell-mediated cellular response Spike-specific of SARS-CoV-2 following prime boost vaccination in naïve and individuals recovered from COVID-19. **(A)** Experimental design of the *ex vivo* T cell-mediated cellular responses in PBMCs, to address early and late responses to the second and third (boost) vaccination, after stimulation with the SARS-CoV-2 wild-type (WT) Spike peptide pool. **(B)** TNFα, IL-6, IL-2, IFNγ, CXCL10, IL-1β, IL-17A, and IL-10 production in naïve participants and recovered from COVID-19 at the indicated sample numbers. **(C)** Increment of proliferation (CFSE^dim^) comparing SARS-CoV-2 Spike peptide pool-stimulated and non-stimulated CD4^+^ (left panel) and CD8^+^ (right panel) T cells in naïve participants and those recovered from COVID-19 at the indicated sample numbers. Data in **(B, C)** are shown as mean ± min to max. Each dot represents a single participant. Unpaired Student’s *t-*test or Mann–Whitney test according to normality, comparing naïve and subjects recovered from COVID-19 at each sample (*#p* < 0.05). Paired Student’s *t-*test or Wilcoxon test according to normality, comparing samples along the time in naïve subjects and those recovered from COVID-19 (*p < 0.05; **p < 0.01; ***p < 0.001; ****p < 0.0001). In every plot, all the possible experimental conditions were compared; statistically significant differences are indicated, and the lack of statistical indication means the absence of a significant difference.

**Figure 3:**
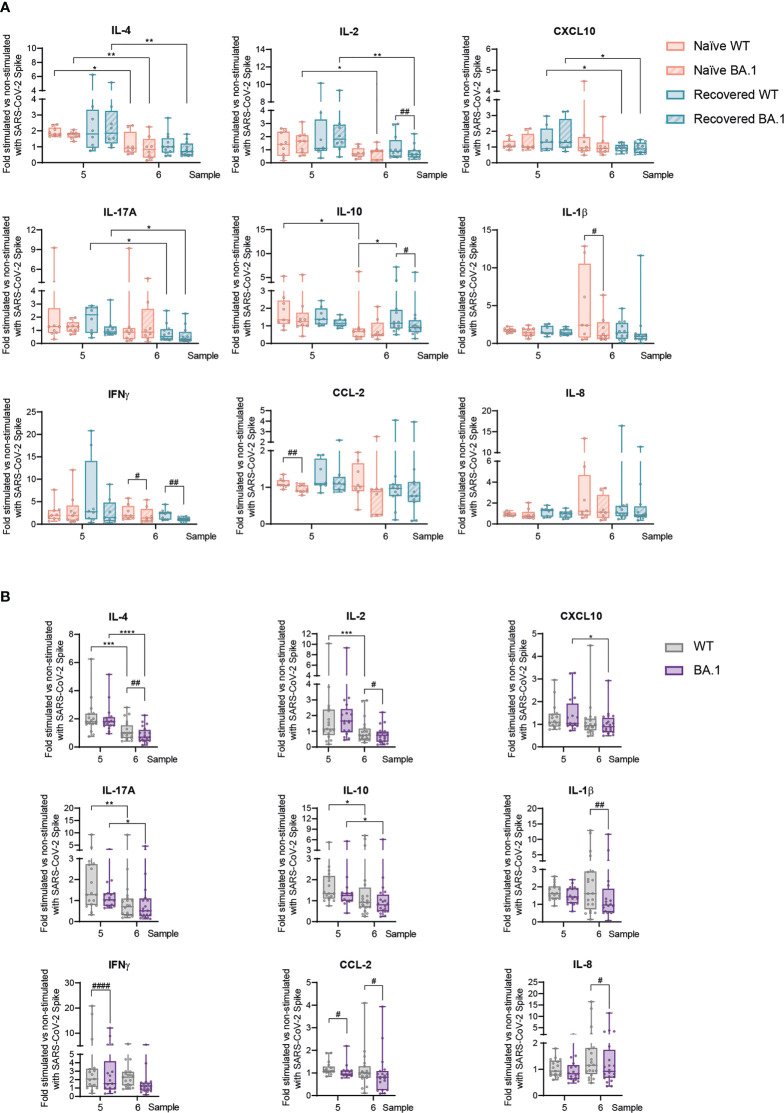
T-cell *ex vivo* responses against SARS-CoV-2 Spike wild type and Omicron BA.1 following prime-boost vaccination in naïve individuals and those recovered from COVID-19. Following the scheme shown in **Figure 2A**, responses to the third vaccination dose, both early and late, were studied in response to either the SARS-CoV-2 wild-type (WT) or Omicron BA.1 variant Spike peptide pool. **(A)** IL-4, IL-2, CXCL10, IL-17A, IL-10, IL-1β, IFNγ, CCL-2, and IL-8 production in naïve participants and those recovered from COVID-19 at the indicated sample numbers and Spike variant. **(B)** IL-4, IL-2, CXCL10, IL-17A, IL-10, IL-1β, IFNγ, CCL-2, and IL-8 production in pooled participants at the indicated sample number. Data are shown as mean ± min to max. Each dot represents a single participant. **(A)**, paired Student’s *t-*test or Wilcoxon test according to normality, comparing samples 5 and 6 in naïve subjects and those recovered from COVID-19 (*p < 0.05; **p < 0.01), or WT *vs*. BA.1 inside each group of participants (*#p* < 0.05; #*#p* < 0.01). **(B)**, unpaired Student’s *t-*test or Mann–Whitney test according to normality, comparing samples 5 and 6 (*p < 0.05; **p < 0.01; ***p < 0.001; ****p < 0.0001), or WT *vs*. BA.1 inside each participant group (*#p* < 0.05; #*#p* < 0.01; ###*#p* < 0.0001). In every plot, all the possible experimental conditions were compared; statistically significant differences are indicated, and the lack of statistical indication means the absence of a significant difference.

Continuous measurements are expressed as mean ± SEM. To assess the normality distribution to all studied variables, a D’Agostino and Pearson normality test was performed. For two-group comparisons of quantitative variables, a Student’s *t-*test either unpaired (*t-*test or Mann–Whitney *U*) or paired (*t-*test or Wilcoxon) was performed, and for multiple-group comparisons of quantitative variables, an ANOVA or Kruskal–Wallis *H* was performed. For all figures, *p*-values are shown as ns: non-significant, *p < 0.05, **p < 0.01, ***p < 0.001, ****p < 0.0001; *#p* < 0.05, #*#p* < 0.01, ##*#p* < 0.001, ###*#p* < 0.0001. Note that in every plot of **Figures 2** and **3**, all the possible experimental conditions were compared; statistically significant differences are indicated, and the lack of statistical indication means the absence of a significant difference.

## Results

### Boost mRNA vaccination (third dose) equalizes titers of SARS-CoV-2-specific antibodies between naïve and COVID-19-recovered individuals

After receiving the BNT162b2 two-dose vaccination schedule recommended by both the FDA and EMA (5), in this work we have explored the SARS-CoV-2-specific responses triggered by the boost with the COVID-19 vaccine mRNA-1273 in this well-controlled cohort. Following the two BNT162b2 doses, both early (14 days) and late (230 days ≈ 8 months) responses were addressed, labeled as samples 2 and 3, respectively (5). 69 days afterward, meaning 320 days since the starting point of the study, sample 4 was obtained to monitor the steady-state status 5 days before receiving the mRNA-1273 boost (**Figure 1A**). Mirroring the schedule followed for the two first doses, 14 days after the third jab, blood samples were obtained (sample 5) to study early responses. Eventually, 189 days (≈ 6 months) post-boost, a new sample was analyzed (sample 6) to monitor late responses to this third vaccination dose (**Figure 1A**). This scheme represents a longitudinal study for almost 1.5 years since the beginning of the vaccination, exploring SARS-CoV-2-specific responses in the same cohort of individuals (5). Hence, a total of 27 individuals received the mRNA-1273 boost. Among them, there was an almost 50% distribution between those that had not been previously exposed to SARS-CoV-2 infection (naïve) and those that had recovered from COVID-19 (**Figure 1B**). Nonetheless, the analysis of antibodies against SARS-CoV-2 N protein (data not shown) indicated a slight rise in the proportion of recovered participants in samples 4 and 5, which was further increased when analyzing late responses to mRNA-1273 boost in sample 6 (**Figure 1B**). Still, differential responses between individuals exposed or not to SARS-CoV-2 infection could be analyzed.

First, we continued the analysis of humoral responses to vaccination by means of plasma SARS-CoV-2-specific immunoglobulin quantification (**Figure 1C**). Shadowed results represent the already known evolution of such antibodies after the two initial doses of the BNT162b2 vaccine (5). More than 2 months after the last analysis and right before the mRNA-1273 boost, the levels of all antibodies dropped slightly, but the presence of higher titers in COVID-19-recovered individuals was still present (**Figure 1C**). As expected, the vaccination boost increased notoriously the concentrations of all analyzed antibodies. Interestingly, early after this boost (sample 5), the levels of antibodies were comparable with those obtained following the first vaccination jab, suggesting a maximum capacity of production. Eventually, the analysis of this humoral responses 6 months after boost showed that IgA titers were essentially negligible, whereas IgG dropped till baseline levels detected before the boost (**Figure 1C**). Of note, the levels of antibodies against SARS-CoV-2, both early and late after the third vaccination dose, were equivalent between naïve and subjects recovered from COVID-19 (**Figure 1C**).

### mRNA-1273 boost maintains the SARS-CoV-2-specific T-cell responses triggered by the second vaccination dose

Next, SARS-CoV-2-specific T-cell responses were evaluated. Peripheral blood mononuclear cells (PBMCs) from both naïve and COVID-19-recovered participants were *ex vivo* stimulated with a peptide pool including the SARS-CoV-2 spike protein from the wild-type (WT) viral variant, hereafter called the S-peptide. Antigen-specific responses were evaluated in terms of cytokine production and T-cell proliferation (**Figure 2A**). Stimuli were performed for 5 days to allow T-cell proliferation (**Supplementary Figure 1**).

Among the cytokines analyzed in response to S-peptide, some of them such as TNFα, IL-6, IL-2, IFNγ, or CXCL10 were differentially produced between naïve subjects and subjects recovered from COVID-19 early after the second vaccination dose (sample 2), with higher levels in the former group. However, following this early response, no further differences were found due to the SARS-CoV-2 infection between naïve and COVID-19-recovered individuals along the study (**Figure 2B**).

Then, we analyzed the longitudinal behavior of those cytokines induced in response to S-peptide stimulation (**Figure 2B**), as some of them were not produced in an antigen-specific manner (**Supplementary Figure 2**). Worthy of note is that SARS-CoV-2-specific T-cell responses following the second vaccination dose dropped along the time, with the only exception of IL-1β production late after the boost jab (**Figure 2B**).

The parallel analysis of T-cell proliferation corroborated this pattern. S-peptide induced CD4^+^ T-cell proliferation to a higher extent in naïve than in subjects recovered from COVID-19 early after the second vaccination dose, with a similar trend in CD8^+^ T cells (**Figure 2C**). This differential response not only disappear along the time but also showed a light decreasing longitudinal pattern (**Figure 2C**). Importantly, the vaccination boost maintained the SARS-CoV-2-specific T-cell-mediated proliferation, with a slight decrease late after boost in recovered individuals (**Figure 2C**). The analysis of T-cell memory populations comparing samples 5 and 6 after boosting suggests a more dynamic behavior in COVID-19-recovered individuals, with reduced central memory CD4^+^ T and naïve CD8^+^ cells and a concomitant increase in effector memory CD8^+^ T cells (**Supplementary Figure 3**).

Therefore, since late after the second vaccination dose onward, SARS-CoV-2-specific T-cell responses got mostly balanced between naïve individuals and subjects that had recovered from COVID-19. Furthermore, these responses triggered by the second vaccination dose were essentially maintained or slightly decreased equally in both groups along the time.

### Dampened SARS-CoV-2-specific T-cell responses against the Omicron BA.1 variant are comparable between naïve and COVID-19-recovered subjects

Next, we wanted to evaluate SARS-CoV-2-specific T-cell responses after the booster jab against the Omicron BA.1 variant of concern, as the vaccination schedule with this third dose coincided temporally with the rise of this variant. Note that, due to the large mutation load contained in the Spike BA.1 variant (21), the WT pool of peptides used in here is different from previous experiments and represents the actual WT reference for the Spike BA.1 variant. Similarly, as observed previously at late time points (**Figure 2**), the WT S-peptide-specific responses showed a limited strength for most of the analyzed cytokines, with essentially no differences between naïve individuals and subjects recovered from COVID-19, along with a decreasing profile between early (sample 5) and late (sample 6) responses (**Figure 3A**). Importantly, for some of the analyzed cytokines such as IL-2, IL-10, IL-1β, IFNγ, and CCL-2 (**Figure 3A**) and even for cytokines not induced after S-peptide stimulation such as IL-6 or TGFβ (**Supplementary Figure 4A**), the Omicron BA.1 pool of peptides generated a poorer response in sample 6. These data suggested that this variant of concern triggered dampened T-cell-mediated responses, in particular late after the boost vaccine dose. Considering the equivalent responses between naïve subjects and those recovered from COVID-19 following the booster vaccination, we decided to analyze the readouts against Omicron BA.1 combining both groups of participants, in an attempt to have a more robust information about the responses triggered by this variant of concern. This analysis reinforced the drop in T-cell-mediated responses late (sample 6) after the third vaccination dose (**Figure 3B**). At the same time, it also showed that the responses to Omicron BA.1 were lower than its corresponding WT. Interestingly, this was more remarkable at the late time point (sample 6) after the vaccination boost, with five cytokines (IL-4, IL-2, IL-1b, IL-8, and TGFβ) out of seven differentially expressed between WT and BA.1 showing this temporal pattern (**Figure 3B** and **Supplementary Figure 4B**). T-cell proliferation was also analyzed in these contexts, but most likely due to the limited activation induced by S-peptides in these late samples, no statistically significant differences were detected (**Supplementary Figure 5**). 

### Omicron BA.1 variant generates reduced functional responses in B cells

**Figure 4:**
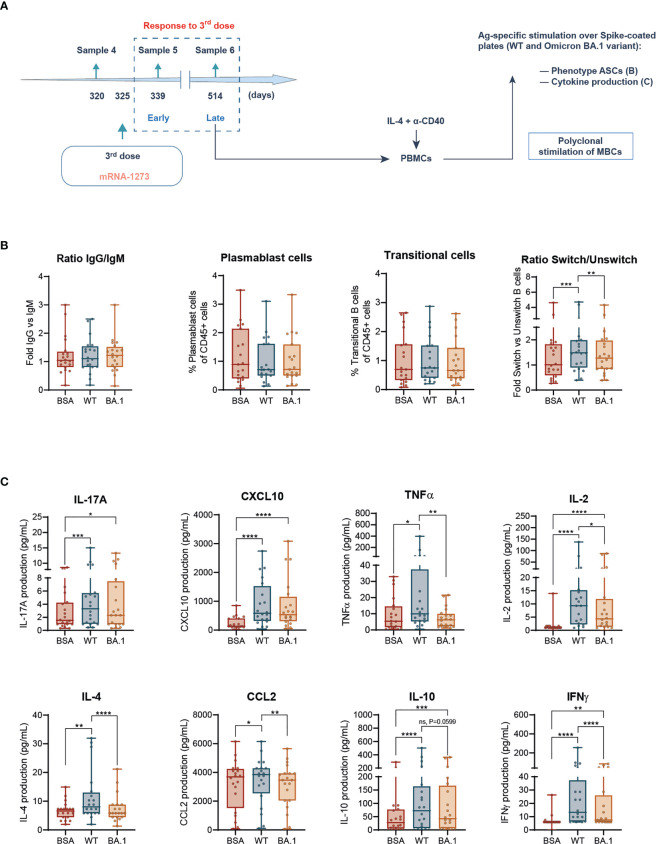
B-cell *ex vivo* responses against SARS-CoV-2 Spike wild type and Omicron BA.1 following prime-boost vaccination. **(A)** Experimental design of the B cell-mediated cellular responses *ex vivo* in PBMCs, after polyclonal stimulation of memory B cells (MBCs) with IL-4 + anti-CD40, to address late responses (sample 6) to the third (boost) vaccination dose. Following this step, generated antibody-producing cells were counted and plated on Spike-coated plates, either the WT or Omicron BA.1 variant of concern. BSA-coated plates were used as negative control. **(B)** Ratio of IgG/IgM in IgD- memory B cells, frequency of CD27^+^ CD20^-^ plasmablasts, and IgD^-^CD38^+^ transitional cells and ratio between class switched versus unswitched CD19^high^ CD20^high^ cells. **(C)** IL-17A, CXCL10, TNFα, IL-2, IL-4, CCL-2, IL-10, and IFNγ production in pooled participants following stimulation with plated BSA, or SARS-CoV-2 Spike protein, either WT or Omicron BA.1. Data are shown as mean ± min to max. Each dot represents a single participant. **(B, C)**, paired Student’s *t-*test or Wilcoxon test according to normality, comparing stimuli (*p < 0.05; **p < 0.01; ***p < 0.001; ****p < 0.0001). In every plot, all the possible experimental conditions were compared; statistically significant differences are indicated, and the lack of statistical indication means the absence of a significant difference.

Due to the relevance of humoral responses against SARS-CoV-2 infection and considering that the titers of specific antibodies late after the vaccination boost (sample 6) were dramatically reduced (**Figure 1C**), we decided to explore the functionality of B cells after reexposing them *ex vivo* to Spike protein, both WT and Omicron BA.1 variants of concern. In this sense, it is important to consider the dampened reactions observed in T-cell activity against the Omicron BA.1 variant of concern. Considering the apparent power of our experimental approach to detect differential behaviors by analyzing the production of cytokines (**Figure 3**), we decided to address SARS-CoV-2-specific functional responses in B cells against both WT and BA.1 variants using this methodology. First, we generated antibody-secreting cells (ASCs) from PBMCs by polyclonal stimulation of memory B cells (MBCs) with IL-4 and anti-CD40. Then, these cells were stimulated in an antigen-specific manner with plate-coated Spike protein, either WT or Omicron BA.1, analyzing the phenotype of the resulting ASCs and their cytokine production (**Figure 4A**).

Following the analysis described for B cells in a full-spectrum flow cytometry (22) (**Supplementary Figure 6A**), we observed that the stimulation with Spike protein either WT or Omicron BA.1 was innocuous in the IgG/IgM ratio and frequency of plasmablast or transitional cells (**Figure 4B**). However, WT Spike induced a class switch in ASCs that was absent in BA.1-stimulated cells (**Figure 4B**). Therefore, this analysis suggested a dampened response to the Omicron variant also in terms of B-cell activation.

We next analyzed the cytokine production by these cells in response to both Spike protein variants. Following the same pattern observed for class-switching, the production of nearly all the induced cytokines in response to the WT Spike protein (TNFα, IL-2, IL-4, CCL2, IL-10, and IFNγ) was reduced or even absent in response to Omicron BA.1 Spike protein (**Figure 4C**). This was also the case for IL-8 among the not-induced cytokines due to stimulation (**Supplementary Figure 6B**).

Altogether, these data indicate that functional responses of B cells against the Spike protein from the SARS-CoV-2 Omicron BA.1 variant of concern are dampened compared with those triggered in response to the Spike WT.

## Discussion

This work represents a longitudinal study of responses triggered by a heterologous prime-boost vaccination scheme with mRNA vaccines against COVID-19, for almost 1.5 years. The study includes a comprehensive survey of immunological features including humoral responses in terms of circulating SARS-CoV-2-specific antibodies, T-cell-mediated reactions after *ex vivo* SARS-CoV-2-specific re-stimulation, as well as the analysis of B-cell-based cellular responses after polyclonal differentiation of antibodies secreting cells from PBMCs, and subsequent *ex vivo* SARS-CoV-2-specific restimulation, analyzing the phenotype of the differentiated cells and their cytokine production profile. All these parameters have been analyzed comparing the behavior observed in naïve subjects and participants recovered from COVID-19, also including the comparative study of responses triggered by the original wild-type (WT) version of the SARS-CoV-2 Spike protein and the Omicron BA.1 variant of concern (VoC). Worthy of note is that this study has been performed based on a cohort of healthy health workers, all vaccinated at the same time, with the same vaccines and vaccination schedule, and including only COVID-19-recovered individuals that suffered from mild disease, who did not require clinical intervention. Therefore, considering the impact of different determinants on the immunological responses to SARS-CoV-2 infection and COVID-19 vaccination (23, 24), this constitutes a well-standardized cohort. To the best of our knowledge, this represents a unique integral immunological study performed on the basis of such a long longitudinal scheme, addressing SARS-CoV-2-specific responses after prime-boost COVID-19 vaccination.

Alternative longitudinal studies support some of the information provided by our work, nevertheless segmented. Most of the studies focused their longitudinal findings in the quantification of circulating SARS-CoV-2-specific antibodies, mainly addressing the differential responses triggered against WT and Omicron Spike proteins. This is consistent with our design, as booster vaccination doses were contemporary to the rise of the Omicron BA.1 VoC (World Health Organization, 2022). Still, for most of these studies, the longitudinal duration is limited compared with ours, with analyses following boost vaccination for 1 month (25–27), 4 months (28), or 6 months (29), the latter in a comparable scheme to our study. The longest study that we have found lasted for 8 months following the booster dose, analyzing the efficacy of a rapid lateral flow assay to detect antibodies against the SARS-CoV-2 Spike receptor binding domain (RBD) (30). Importantly, none of these studies differentiate between naïve and participants recovered from COVID-19. Our data indicate that until receiving the booster vaccine dose, humoral responses were stronger in those individuals with a previous infection with SARS-CoV-2. On the other hand, early T-cell responses after the two first doses were dampened in COVID-19-recovered individuals, which might be associated with the generation of immunomodulatory T regulatory cells (5) and/or T-cell exhaustion due to SARS-CoV-2 infection, as described elsewhere (31–33). Nevertheless, T-cell-mediated responses were comparable in both naïve and recovered participants following 7 months after the second vaccine jab. Therefore, considering a previous infection by SARS-CoV-2 is relevant to fully understanding the effects triggered by prime-boost COVID-19 vaccination schedules.

The observed differential behaviors between humoral and T-cell-mediated responses are consistent with former studies. The booster effect of the third vaccination dose on the production of SARS-CoV-2-specific antibodies is key to supporting the recommendation for this recall vaccination. Furthermore, those studies analyzing in parallel humoral and T-cell responses concur with our data regarding the stable behavior of cellular reactions late after two vaccination doses (20, 26). Whether this effect has something to do with certain T-cell exhaustion requires further deeper analyses, as this effect has been mostly studied in the context of acute and severe SARS-CoV-2 infections (32, 34). As T-cell activation has been shown to correlate with reinfection (35), and infection is not rare in vaccinated populations, it is tempting to speculate that vaccine-triggered humoral responses are fundamental for the protection versus severe COVID-19, whereas cellular reactions are more decisive for reinfection, although both systems work in a concerted manner. However, this interpretation could be misleading considering that this analysis is performed during waves of different virus strains that might differentially impact both branches of immunity (36).

Along these lines, the rise of the Omicron BA.1 VoC threated the *status quo* generated by the initial two-dose vaccination schedules, due to the heavy load of mutations carried by this variant in the Spike protein and in the RBD specifically (37), which is a major target for neutralizing antibodies generated after vaccination. Very early after its characterization, it was clear that Omicron BA.1 had the capacity to escape from neutralizing antibodies (38), although vaccination was still partially efficient against this variant (39). Our data and the abovementioned longitudinal studies (27–29) corroborated this fact. However, our study provides an extra layer of complexity with the analysis of cellular functions triggered by antibody-secreting cells (ASCs). Functional interrogation *ex vivo* of these cells represents a powerful tool to identify patterns of response *in vivo* (40). Nevertheless, knowledge about functionality of B cells after specific restimulation beyond detection of SARS-CoV-2-specific BCR-expressing cells (26, 41) is scarce, in particular after such a long period following the booster vaccination, and in response to the Omicron VoC. In line with these results, no differences were found in T-cell proliferation but detected in cytokine production after T-cell activation following the booster vaccination. Furthermore, antibody-independent functions of B cells, mainly cytokine production, can play essential roles in homeostasis, activation of lymphoid organs, and development of T-cell responses (42), with relevance as therapeutic targets (43). These results highlight the relevance of extending the range of functional readouts to fully address differential responses, even in conditions of low strength activation. Thus, it appears critical to perform SARS-CoV-2-specific activation approaches to uncover functional information, beyond the characterization of antigen-specific receptor-expressing cells.

Based on these functional analyses, we have confirmed that responses triggered by the Spike protein from the Omicron BA.1 (B.1.1.529/BA.1) VoC are attenuated compared with its reference WT. These results go in line with the current knowledge (20, 26) and has prompted the recommendation of a second vaccine boost, in particular for patients endangered for severe COVID-19 such as patients receiving hemodialysis (25) or transplant recipients (44).

Summarizing, this work indicates that a previous infection with SARS-CoV-2 modulated responses to COVID-19 vaccination after the two-dose prime phase. Booster vaccination balanced these responses between naïve and subjects recovered from COVID-19. Responses triggered against the SARS-CoV-2 Omicron BA.1 VoC are dampened compared with WT virus, and after receiving a booster vaccination dose, these responses are equally diminished independently of a previous SARS-CoV-2 infection. This fact applies to both T-cell and B-cell cellular responses. Therefore, a fourth booster vaccination dose is highly recommended, where variant-specific vaccines should be considered.

## Data availability statement

The raw data supporting the conclusions of this article will be made available by the authors, without undue reservation.

## Ethics statement

The studies involving human participants were reviewed and approved by CEIm “La Paz University Hospital” (PI-4100). The patients/participants provided their written informed consent to participate in this study.

## Author contributions

EL-C, CdF, RL-R, and JA-O designed the study. CdF wrote the manuscript. AM-Q, AB-C, MG-G, MP, IL-F, LG, GM-M, and CH-B recruited the participants and collected the samples. RL-R, JA-O, VT, KH, JC-D, MB-G and PM-M performed the experiments. LL-M and CV-O provided critical experimental data during revision of the manuscript. EL-C, CdF, RL-R, JA-O and CC-Z discussed the results. All authors contributed to the article and approved the submitted version.
