# Supplementary material for: mRNA-1273 boost after BNT162b2 vaccination generates comparable SARS-CoV-2-specific functional responses in naïve and COVID-19-recovered individuals
Source: Front Immunol. 2023 Apr 21;14:1136029. doi: 10.3389/fimmu.2023.1136029 (PMC10160618; doi:10.3389/fimmu.2023.1136029)
Supplement: Supplementary file 1 [file DataSheet_1.pdf]

Supplementary Material

Supplementary Figures

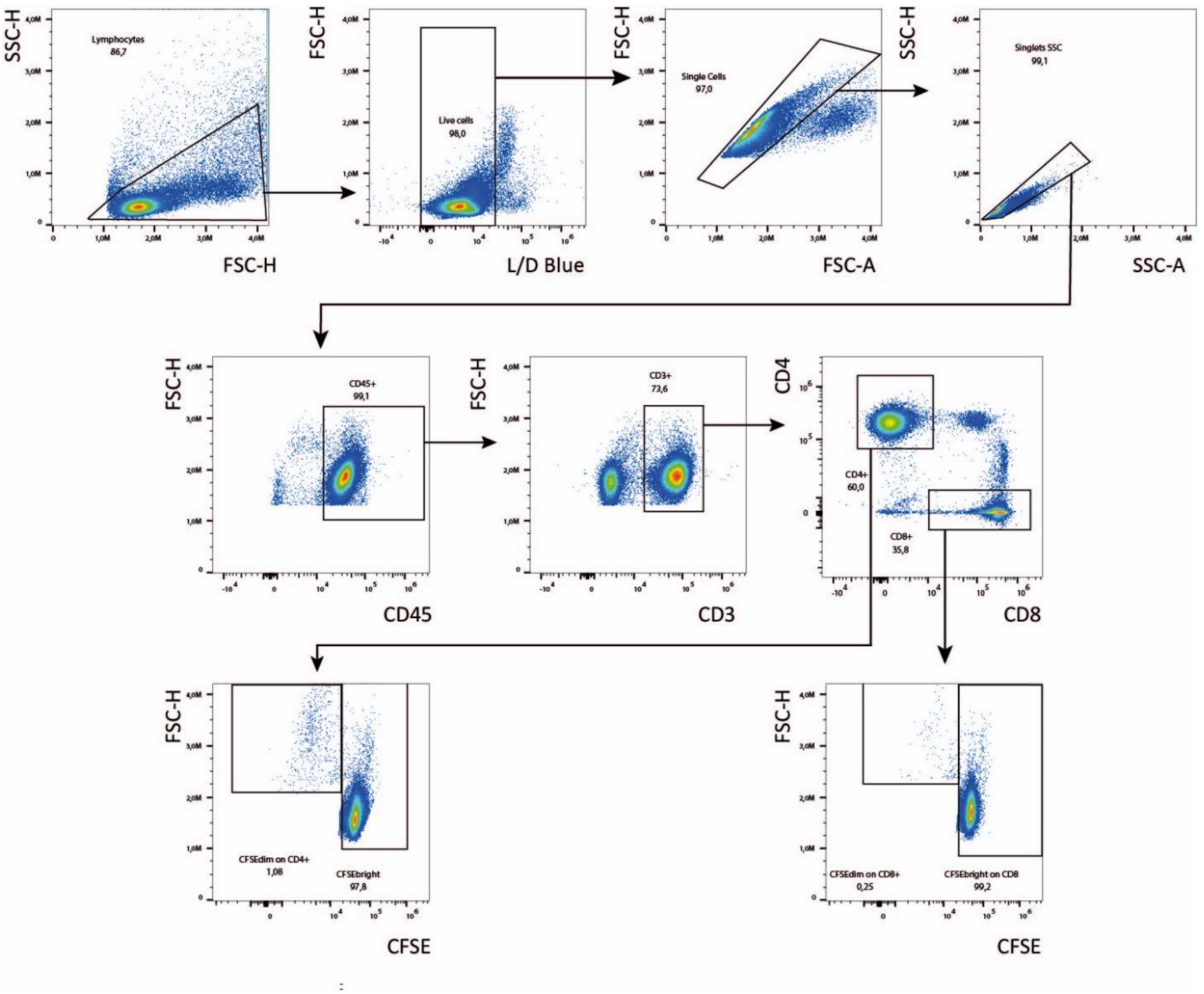

Supplementary Figure 1. FACS template used to analyze SARS-CoV-2 Spike-specific T cell responses.

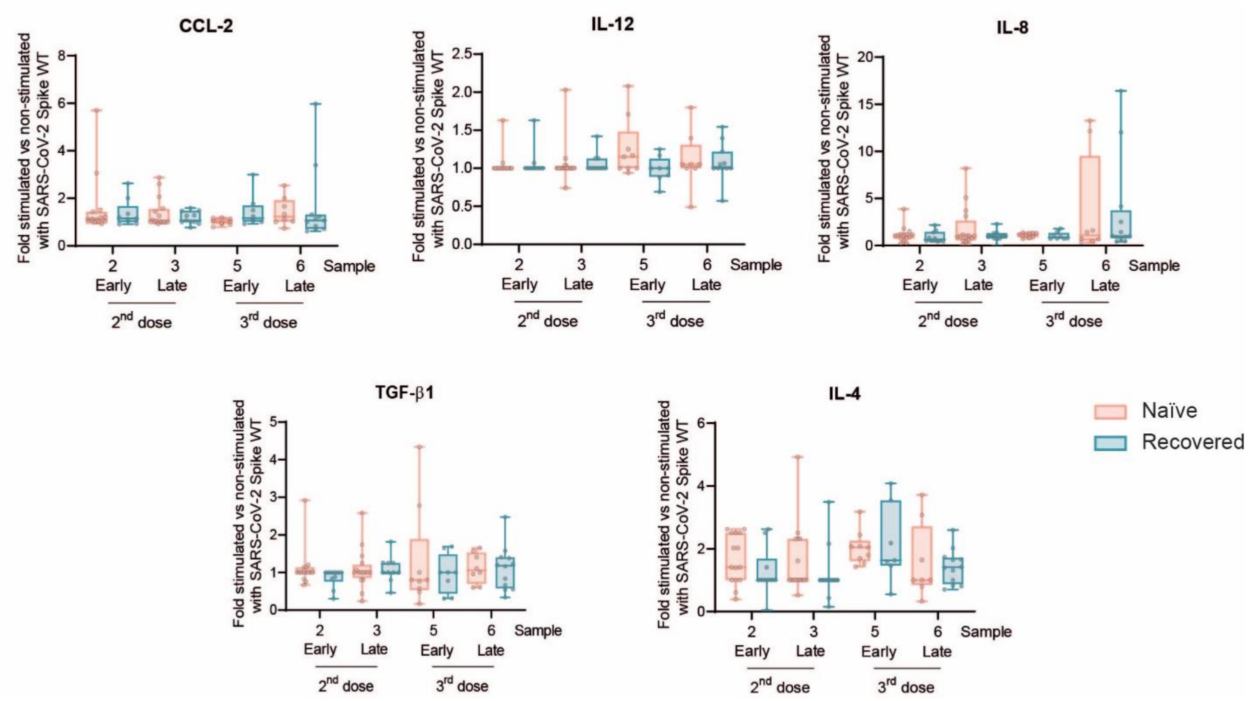

Supplementary Figure 2. SARS-CoV-2 Spike-specific T cell-mediated *ex vivo* responses following prime-boost vaccination in naïve and individuals recovered from COVID-19. CCL-2, IL-12, IL-8, TGF-β1 and IL-4 production in naïve participants and recovered from COVID-19 at the indicated sample numbers. Cytokines not induced in sample 2 are shown.

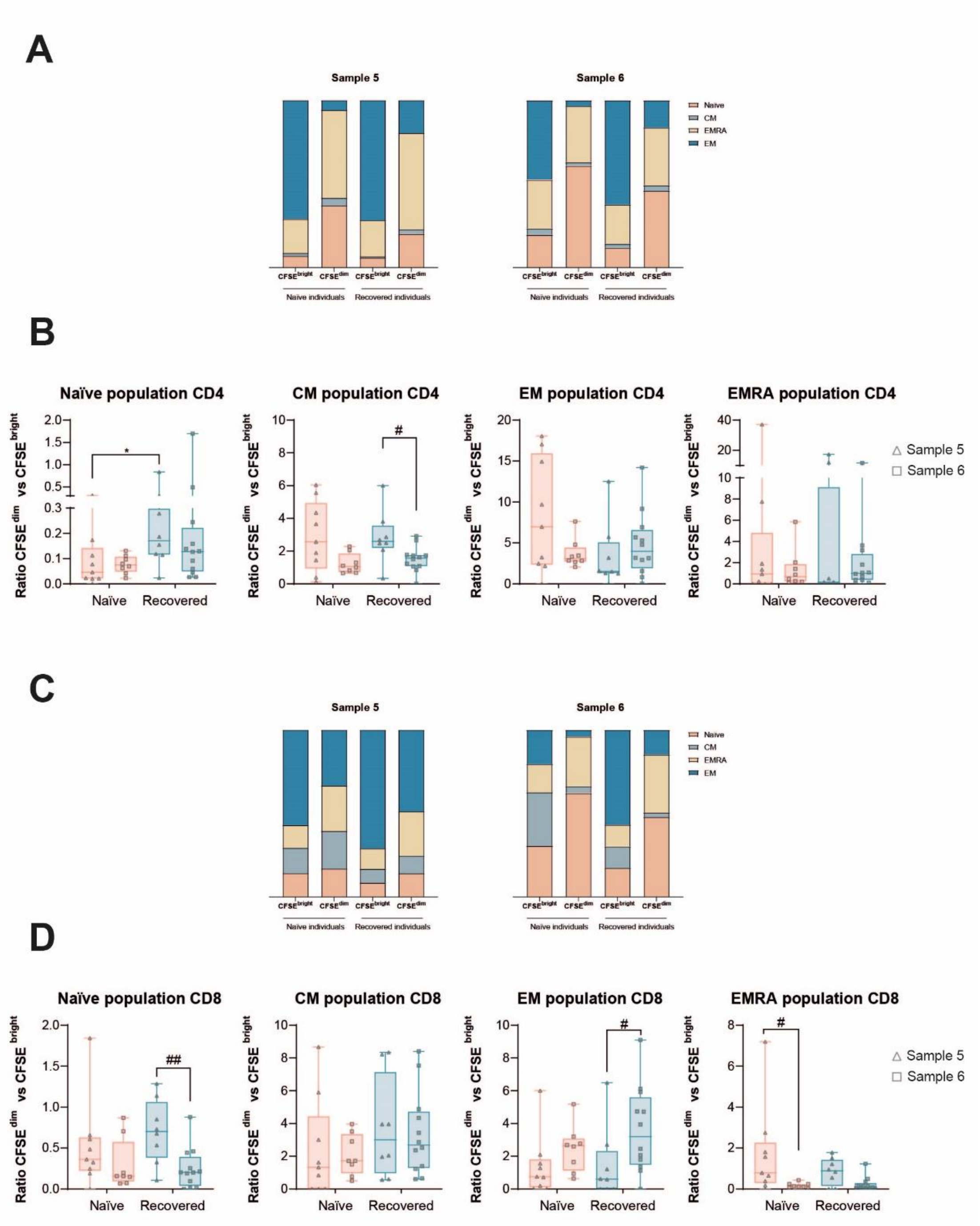

Supplementary Figure 3. Memory subpopulations in SARS-CoV-2 spike-specific $CD4^{+}$ and $CD8^{+}$ T cells in naive subjects and individuals recovered from COVID-19. Memory subpopulations (naïve; CM, central memory; EMRA, effector memory cells re-expressing CD45RA; EM, effector memory) were analyzed in T cells. **(A)** Frequency of memory populations in proliferative ($CFSE^{dim}$) and non-proliferative ($CFSE^{bright}$) $CD4^{+}$ T cells. **(B)** Proliferative ($CFSE^{dim}$) versus non-proliferative ($CFSE^{bright}$) ratio of $CD4^{+}$ T cell memory populations in samples 5 (Δ) and 6 (□). **(C)** Frequency of memory populations in proliferative ($CFSE^{dim}$) and non-proliferative ($CFSE^{bright}$) $CD8^{+}$ T cells. **(D)** Proliferative ($CFSE^{dim}$) versus non-proliferative ($CFSE^{bright}$) ratio of $CD8^{+}$ T cell memory populations in samples 5 (Δ) and 6 (□).

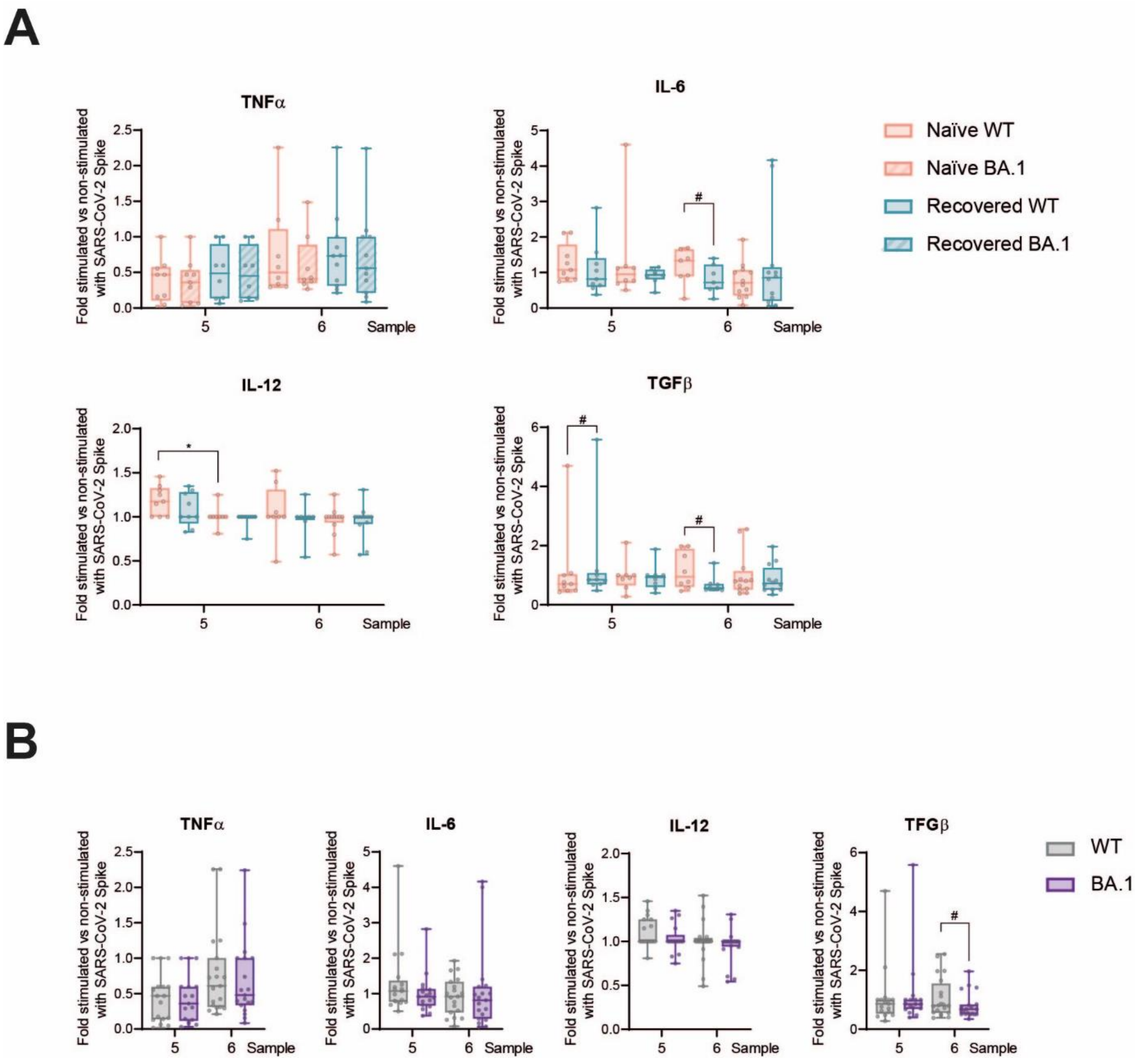

Supplementary Figure 4. T cell *ex vivo* responses against SARS-CoV-2 Spike wild type and Omicron BA.1 following prime-boost vaccination in naïve and individuals recovered from COVID-19. Following the scheme shown in figure 2A, responses to the third vaccination dose, both early and late, were studied in response to either SARS-CoV-2 wild type (WT) or Omicron BA.1 variant Spike peptide pool. **(A)** TNFα, IL-6, IL-12 and TGFβ production in naïve participants and recovered from COVID-19 at the indicated sample numbers and Spike variant. **(B)** TNFα, IL-6, IL-12 and TGFβ production in pooled participants at the indicated sample number. A, paired Student's t-test or Mann Whitney test according to normality, comparing samples 5 and 6 in naïve and subjects recovered from COVID-19 ($^{*}p < 0.05$), or WT vs BA.1 inside each group of participants ($^{\#}p < 0.05$). B, paired Student's t-test or Wilcoxon test according to normality, comparing WT vs BA.1 inside each participant group ($^{\#}p < 0.05$). Cytokines not induced in sample 5 are shown.

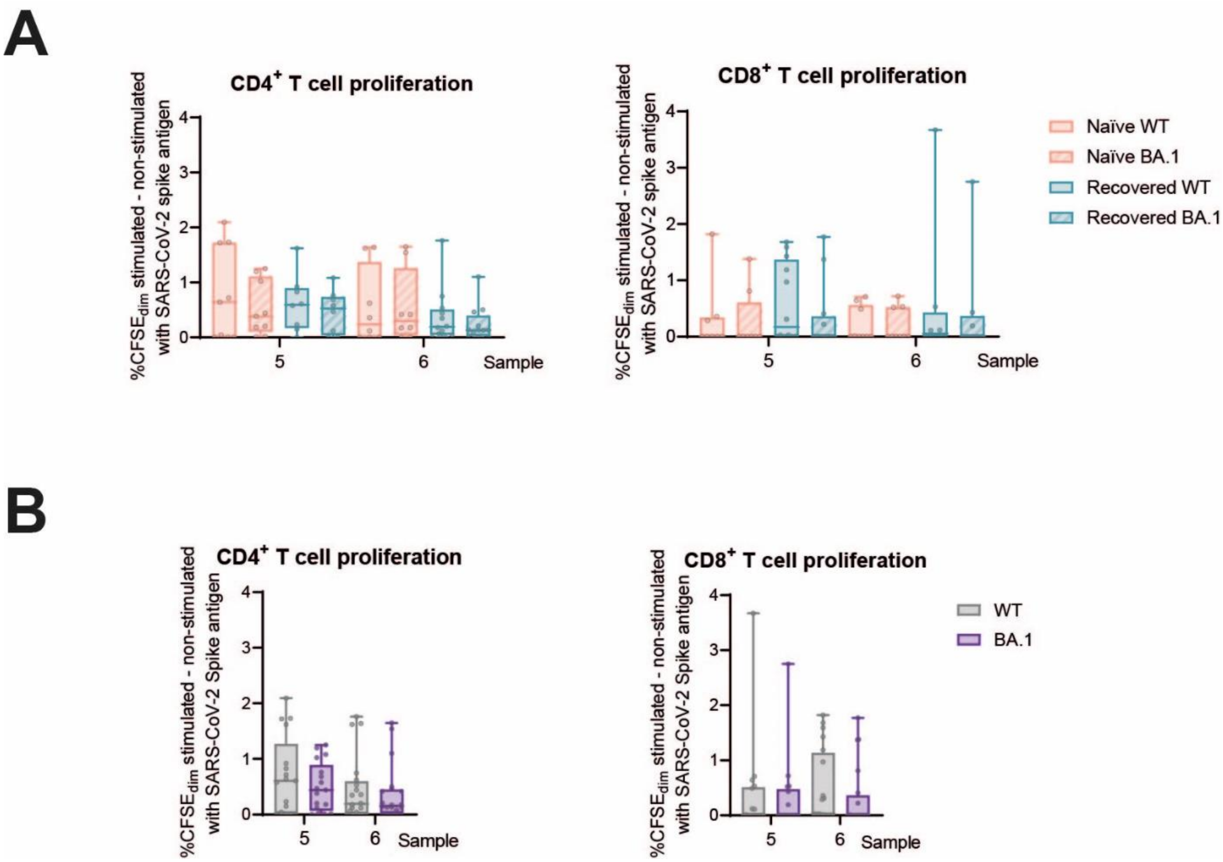

Supplementary Figure 5. T cell proliferation against SARS-CoV-2 Spike wild type and Omicron BA.1 following prime-boost vaccination. Following the scheme shown in figure 2A, T cell proliferation was analyzed as follows the increment of proliferation ($CFSE^{dim}$) comparing SARS-CoV-2 Spike peptide pool-stimulated and non-stimulated $CD4^+$ (left panel) and $CD8^+$ (right panel) T cells in naïve participants and recovered from COVID-19 **(A),** or pooled **(B)** at the indicated sample numbers.

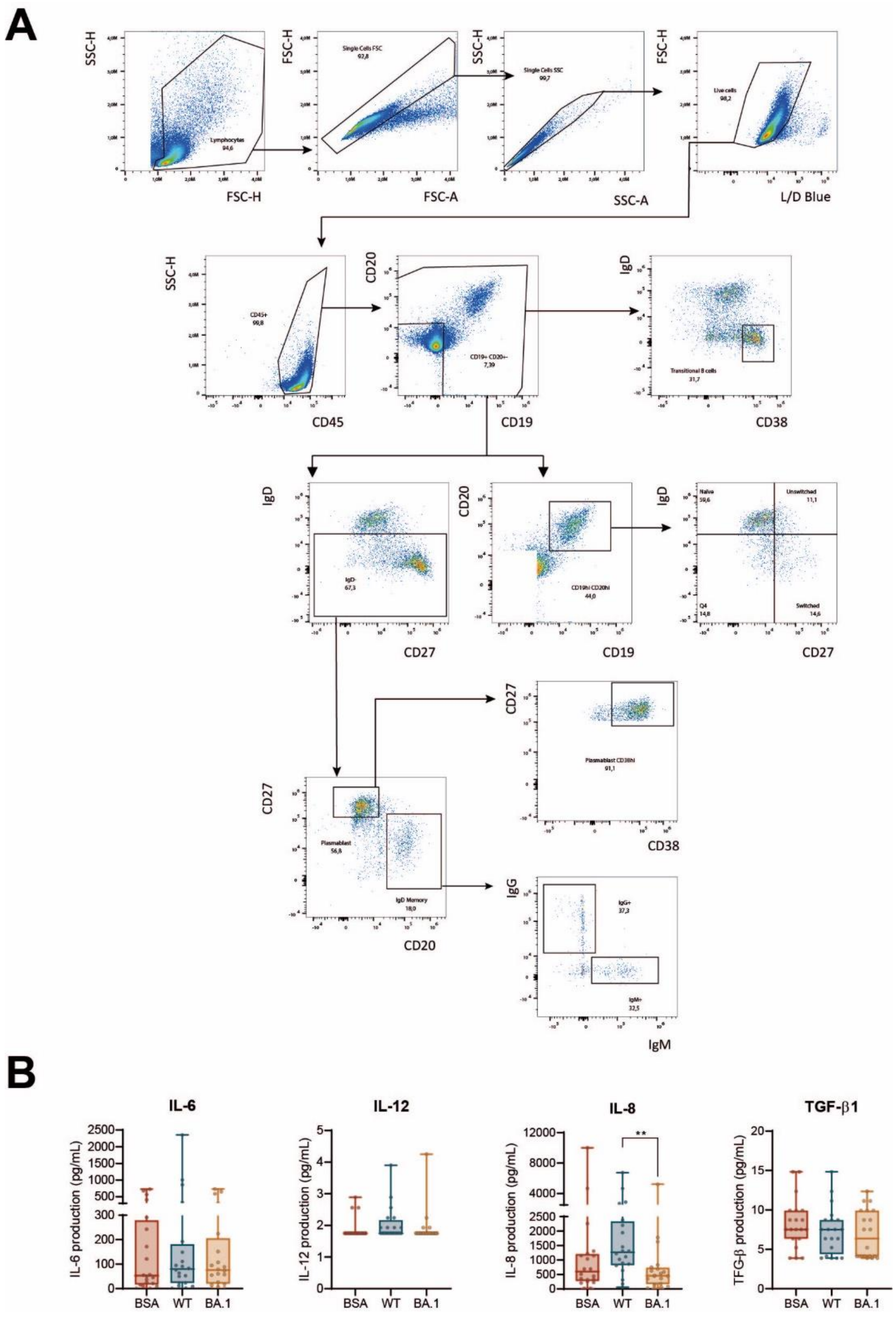

Supplementary Figure 6. Analysis of B cell *ex vivo* responses against SARS-CoV-2 Spike wild type and Omicron BA.1 following prime-boost vaccination. (A) FACS template used to analyze SARS-CoV-2 Spike-specific cellular responses in antibodies secreting cells. IL-6, IL-12, IL-8 and TGF-β1 production in pooled participants following stimulation with plated BSA, or SARS-CoV-2 Spike protein, either WT or Omicron BA.1. B, paired Student's t-test or Wilcoxon test according to normality, comparing stimuli (**$p < 0.01$). Cytokines not induced over BSA control are shown.

Supplementary Tables

Supplementary Table 1. List of fluorochrome-conjugated monoclonal antibodies used for T cell proliferation assays by FACS.

Marker	Fluorochrome	Source	Clone	Reference
CD3	BV510	Biolegend	OKT3	Cat# 317332
CD4	cFluor-YG584	Cytek Biosciences	SK3	Cat# R7-20042
CD8	BUV805	BD	SK1	Cat# 612889
CD45	PerCP	Biolegend	2D1	Cat# 368506
CD45RA	BUV395	BD	5H9	Cat# 740315
CD62L	BV615	BD	SK11	Cat# 751364
CD28	BV650	Biolegend	CD28.2	Cat# 302946
CCR7	BV421	Biolegend	G043H7	Cat# 353208

Supplementary Table 2. List of fluorochrome-conjugated monoclonal antibodies for Spike-specific memory B cell activation by FACS.

Marker	Fluorochrome	Source	Clone	Reference
CD19	Spark NIR 685	Biolegend	HIB19	Cat# 302270
CD20	Pacific Orange	ThermoFisher	HI47	Cat# MHCD2030
CD24	PE/Dazzle 594	Biolegend	ML5	Cat# 311134
CD25	PE-AlexaFluor 700	ThermoFisher	CD25-3G10	Cat# MHCD2524
CD27	APC	Biolegend	M-T271	Cat# 356410
CD38	APC-Fire 810	Biolegend	HIT2	Cat# 303550
CD45	PerCP	Biolegend	2D1	Cat# 368506
IgD	BV480	BD	IA6-2	Cat# 566138
IgM	BV570	Biolegend	MHM-88	Cat# 314517
IgG	BV605	BD	G18-145	Cat# 563246
